# Epidemic hysteria following the National School Deworming Day, Zamboanga Peninsula, Philippines, 2015

**DOI:** 10.5365/wpsar.2017.8.1.009

**Published:** 2018-12-03

**Authors:** Johnette A. Peñas, Vikki Carr de los Reyes, Ma. Nemia L. Sucaldito, Julius Erving D. Ballera, Herdie L. Hizon, Rio L. Magpantay, Vicente Y. Belizario, Kenneth Hartigan-Go

**Affiliations:** a Department of Health, Philippines.

## Abstract

**Introduction:**

In July 2015, the Philippines conducted a school-based mass drug administration using albendazole for soil-transmitted helminths infection. Reports of adverse events were subsequently made through the event-based surveillance system, mostly from the Zamboanga Peninsula on the island of Mindanao. A team from the Epidemiology Bureau investigated the reports of adverse events following mass drug administration (AEFMDA).

**Methods:**

Five schools were identified for the investigation which comprised an unmatched case-control study, key informant interviews and laboratory examinations. AEFMDA cases were students who had sudden onset of abdominal pain, vomiting, diarrhoea, loss of consciousness, headache or dizziness within 24 hours after intake of deworming tablet; controls were healthy students who did not develop signs and symptoms after deworming.

**Results:**

Most (85%) of the 7313 AEFMDA cases reported nationwide were from Zamboanga Peninsula. Most reports were made after rumours of deaths following deworming and of the use of expired drug were spread through the region. Many parents sent their children to hospital, even if asymptomatic. The case-control study found that being an AEFMDA case was associated with no history of previous deworming (odds ratio = 4.08, 95% confidence interval: 1.77–9.42).

**Discussion:**

The investigation concluded that epidemic hysteria was the cause of the increased number of AEFMDA cases in the Zamboanga Peninsula. The false information, aggravated by social media, caused panic and an increase in reporting. Some cases had no history of deworming, and they may not have been aware that albendazole is safe and that side-effects are expected. Risk communication before, during and after future national deworming programmes are recommended to prevent unnecessary reporting of AEFMDA.

## Introduction

Over 2 billion people suffer from soil-transmitted helminths worldwide. (*1*) In the Philippines, soil-transmitted helminths affect all provinces. (*2*) Before the implementation of the National School Deworming Day (NSDD) in 2015, month-long nationwide deworming programmes were administered to preschool-age children (1–4 years old) in the community by City and Rural Health Units, while school-age students (5–18 years old) were dewormed by the Department of Education in public elementary and secondary schools. (*2*, *3*) The prevalence of soil-transmitted helminths has decreased from 66% among children aged 1–5 years old and 65% among children aged 6–14 years old in 2003 to 28.4% in school-aged children in 2013–2015. (*4*, *5*)

Mass deworming programmes in school-age children are recommended by the World Health Organization (WHO). (*1*, *6*) The Philippines Department of Health, in partnership with the Department of Education, conducted the first NSDD on 29 July 2015. (*3*) The NSDD aimed to deworm approximately 16 million school-age children enrolled in all public elementary schools in one day to reduce the burden of soil-transmitted helminths infections. It was anticipated that the NSDD strategy would have a major impact on the Integrated Helminth Control Program accomplishments, and pilot projects in Regions 6 and 11 showed that a one-day deworming programme is feasible and improves the efficiency of service delivery among the target population. (*7*)

On the day of the NSDD, cases of adverse events following mass drug administration (AEFMDA) were reported to the national event-based surveillance system from schools, health centres and hospitals. Most reports were from the Zamboanga Peninsula in Region 9 on the island of Mindanao. Therefore, a team from the Epidemiology Bureau of the Department of Health was sent to Zamboanga Peninsula to investigate the reports of AEFMDA.

## Methods

### Case finding

The Zamboanga Peninsula region had the highest number of reports of AEFMDA in the event-based surveillance system and was therefore selected for the investigation. Due to insurgency and armed conflict in some areas of the region, five schools that had reported cases and were identified as being safe by the Zamboanga Peninsula Regional Epidemiology Surveillance Unit were selected for the investigation.

### Case-control study

A 1:2 unmatched case-control study was conducted in the five schools. A standard questionnaire with closed- and open-ended questions was used to identify sociodemographic risk factors and exposure history. An AEFMDA case was any student from the selected schools on the Zamboanga Peninsula who reported abdominal pain, vomiting, diarrhoea, loss of consciousness, headache or dizziness within 24 hours after intake of deworming tablet on 29 July 2015. A control was any healthy student from the same schools who did not develop any signs and symptoms after receiving the deworming tablet on 29 July 2015. Only those students whose parents and teachers consented to the interview were included in the study.

Cases were classified based on WHO guidelines for degree of severity. (*8*, *9*) Odds ratios (OR), 95% confidence intervals (CI) and *P*-values were calculated using Epi Info version 3.5.4. Risk factors approaching significance (*P* < 0.2) in bivariate analysis were included in a multivariable logistic regression using the backward elimination procedure. Significant level of α = 0.05 and two-tailed *p*-value of the test was used.

### Key informant interviews

Semi-structured interviews were conducted in person with Zamboanga Peninsula health and school personnel who were involved in the conduct of the NSDD. Information about activities before, during and after the NSDD were elicited.

### Laboratory examination

Rectal swabs were collected from cases and sent to the Research Institute for Tropical Medicine for bacteriological culture for *Salmonella, Shigella, Vibrio* and *Staphylococcus* species.

Albendazole samples of the same batch and lot number as the deworming tablets used during the NSDD were collected from schools and health centres and sent to the Food and Drug Administration for testing for the active component.

## Results

Of the almost 12 million children administered deworming tablets during the NSDD, there were 7330 AEFMDA cases reported to the event-based surveillance system (0.06%). Most of these (6236/7330, 85%) were from the Zamboanga Peninsula, giving an incidence proportion of 1.28% (6236/486 490).

### Case-control study

There were 77 cases of AEFMDA identified at the five selected schools included in the case-control study. Their ages ranged from 6 to 16 years (median = 10 years) and 39 (51%) were males. The most affected age group was the 10–14-year-olds. There were 154 controls identified, with the same age and sex distribution as cases (**Table 1**).

**Table 1:** Factors associated with reporting adverse events following the National School Deworming Day, Zamboanga Peninsula, the Philippines, July 2015

Variables	Case (*n* = 77)	Control (*n* = 154)	*P*-value	Crude OR (95% CI)	Adjusted OR* (95% CI)
**Female**	**38**	**76**	**1.00**	**1.00 (0.58–1.73)**	**-**
**0–10 years old**	**47**	**98**	**0.70**	**0.90 (0.51–1.57)**	**-**
**Undernourished**	**5**	**7**	**0.37**	**1.46 (0.45–4.75)**	**-**
**Administered by health-care worker**	**26**	**67**	**0.15**	**0.66 (0.37–1.17)**	**0.69 (0.37–1.28)**
**Reported handwashing before deworming**	**45**	**110**	**0.048**	**0.56 (0.32–0.997)**	**0.58 (0.32–1.06)**
**Received deworming tablet still In** **blister pack**	**29**	**57**	**0.92**	**1.03 (0.58–1.81)**	**-**
**Deworming tablet taken with food**	**65**	**141**	**0.099**	**0.50 (0.22–1.15)**	**0.52 (0.21–1.28)**
**No previous history of deworming**	**17**	**10**	** < 0.01**	**4.08 (1.77–9.42)**	**4.08 (1.77–9.42)**
**History of previous adverse event after deworming**	**0**	**2**	**0.44**	**0.00 (undefined)**	**-**
**Has allergy**	**1**	**1**	**0.56**	**2.01 (0.12–32.63)**	**-**

95% CI = 95% confidence interval; OR = odds ratio

Signs and symptoms of the 77 AEFMDA cases included abdominal pain (95%), headache (47%) and vomiting (34%). Thirty-nine (51%) cases were hospitalized, and there were no deaths reported. There were 11 cases (14%) classified as severe, 20 (26%) as moderate and 46 (60%) as mild. The onset of symptoms ranged from less than 1 to 13 hours after deworming (median = 4 hours). The nutritional status of most cases was normal (91%). Respondents were asked if they had washed their hands before the deworming activity, and 45 (58%) cases reported handwashing. Sixty-five (84%) reported taking the tablet with food, and 17 (22%) had no previous history of deworming.

In the case-control analysis, having no history of previous deworming was associated with being an AEFMDA case (OR = 4.08, 95% CI: 1.77–9.42), whereas the reporting of handwashing was inversely associated with being an AEFMDA case (OR = 0.56, 95% CI: 0.32–0.997). In the multivariable analysis, no history of deworming was the only risk factor associated with being an AEFMDA case (OR = 4.08, 95% CI: 1.77–9.42) (**Table 1**).

### Key informant interviews

A total of 15 personnel were interviewed (five from the health department and 10 from schools). There was no remarkable incidents recorded during the deworming administration other than the chronology of events. The interviewees suggested that there was insufficient orientation in schools and for parents before the NSDD and that parents believed that their children were harmed due to the deworming.

Schools that administered the deworming tablets before 10:00 experienced no problems. It wasn’t until a text message circulated that reported that several students in the region had died due to the deworming activity that the reports of AEFMDA started. Pictures of expired albendazole tablets claimed to have been used during the NSDD were also circulated through social media. It was thought that these rumours resulted in panic at the schools and in the community as parents rushed to the schools and insisted their children be sent to hospital, even those children without symptoms.

**Fig. 1:**
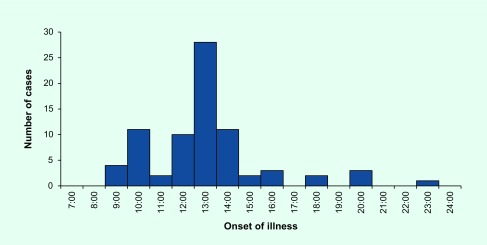
Epidemic curve of cases that reported adverse events following the National School Deworming Day, Zamboanga Peninsula, the Philippines, July 2015 (*n* = 77)

It was also reported that when some children began to report abdominal pain and headache, other children within the same classrooms began to report the same symptoms. In some villages, the village head also announced that all children who received the deworming tablet should go to the hospital. Directly after this, there was a large increase in cases (**Fig. 1**).

### Laboratory examination

Bacteriological culture of rectal swabs collected from 14 cases revealed one (7%) positive for *Vibrio mimicus,* with the remainder negative for all pathogens. All 24 albendazole samples conformed to the drug standard of active component.

## Discussion

We concluded from this investigation that the AEFMDA cases reported in the Zamboanga Peninsula region after the NSDD were primarily due to epidemic hysteria. The high proportion of total AEFMDA cases reported from this region, coupled with the misinformation spread in the community, contributed to the increase of reported cases. The deworming tablets used conformed to drug standards, were used throughout the country and were therefore unlikely to have caused the high number of reported side-effects.

Epidemic hysteria has been defined as a group of symptoms suggestive of organic illness but without identifiable cause. (*10*) Schools are the most common setting for epidemic hysteria outbreak with triggering factors including events and rumours. (*11*) In this investigation, several triggers were identified, including the spread of a text message across the entire region during the NSDD that claimed there were children who died following the intake of the deworming tablet and the circulation of false reports in social media that the deworming tablets used by the Department of Health for the NSDD were expired; both falsehoods were aggravated by media coverage. That insufficient orientation was provided to the schools and parents about the expected side-effects of the deworming tablets also contributed to the increase in reported cases. Epidemics of mass hysteria attract media attention, which usually results in an escalation of such outbreaks. (*12*)

Having no history of previous deworming was significantly associated with AEFMDA cases. The children who had not been dewormed previously and their parents were perhaps less likely to know that taking albendazole is safe and that mild side-effects are expected as they had no experience with the drug. Side-effects occur as the worms pass through a child’s body; mild and moderate adverse reactions are more common after the first dose as children dewormed for the first time are most likely to be heavily infected. (*6*, *13*)

Heavily infected children may experience mild side-effects following deworming, and their reports may trigger other schoolchildren to claim similar symptoms even when they are asymptomatic. (*6*) Mass hysteria following a health intervention has also been reported in schools of Islamic Republic of Iran, Italy, Canada, Jordan and China following vaccinations. (*14*) In 2007, epidemic hysteria occurred in Ghana during a mass elimination campaign of helminths where trained teachers administered mebendazole tablet to nearly 4.5 million children in public schools. Similar to this study, a few hours later, there was news on local radio of deaths due to the programme, which resulted in a wave of unrest and mass hysteria. Such incidents highlight the need for active pharmacovigilance, excellent risk communication and planning of crisis management. (*15*)

During this incident, the Department of Health and Department of Education used risk communication to appease the public. Community assemblies were held and national press releases explaining the NSDD, the potential side-effects of albendazole and the health importance of the programme were disseminated. The public were informed that the medications used during the NSDD were not expired, there had been no deaths related to the deworming tablet and that side-effects are expected, especially in heavily infected children. Risk communication efforts conducted following adverse events after health interventions in Bangladesh, Pakistan, India and Afghanistan have shown to be effective in regaining public trust. (*16*) In March 2016, guidelines on the implementation of the NSDD were amended. (*17*) All school-age children shall be dewormed in one month through the National School Deworming Month.

There are some limitations to this investigation. Psychological testing and assessment of general cognitive abilities of children were not conducted, which may have strengthened the diagnosis of mass hysteria. Another limitation was the unavailability of parasitological data, which may have shown the relationship between reported adverse events and severity of infection. The insurgency and armed conflict in some parts of the region limited the availability of schools to participate in the case-control study, and the small number of respondents may not be generalizable to the whole population of the region. *Vibrio mimicus* was isolated from one case but is unlikely the cause of this event since its incubation period (15–24 hours) (*18*) was not consistent with the event.

Although the reported AEFMDA was low during this deworming programme compared to other studies, the negative impact of the mass hysteria from false reporting may affect the future implementation of the national deworming programme. School and community education efforts that focus on providing a greater understanding of adverse reactions may prevent this and help to achieve the goal of the NSDD. Risk communication before, during and after NSDD in the future is highly recommended.
